# Supplementary figures and images for: Development of a mouse salivary gland-derived mesenchymal cell line for immunological studies of murine cytomegalovirus
Source: PLoS One. 2022 Aug 17;17(8):e0265479. doi: 10.1371/journal.pone.0265479 (PMC9385033; doi:10.1371/journal.pone.0265479)

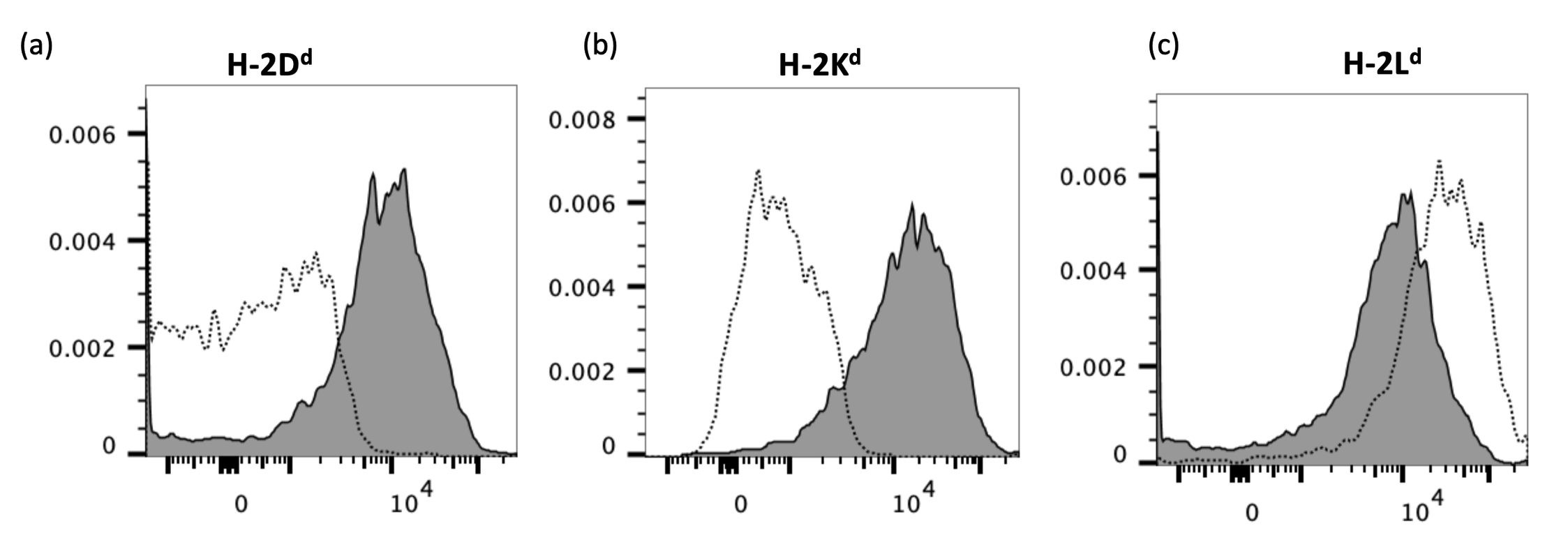

Supplement: S1 Fig — mSGM cells (solid line) and NIH 3T3 cells (dashed line) were stained with antibodies against MHC class I H-2Dd (a), H-2Kd (b), or H-2Ld (c). The antibody for H-2Ld cross-reacts with H-2Db, which is present on NIH 3T3 cells. (TIF) [file pone.0265479.s001.tif]
